# Upper gastrointestinal symptoms, psychosocial co-morbidity and health care seeking in general practice: population based case control study

**DOI:** 10.1186/1471-2296-10-63

**Published:** 2009-09-09

**Authors:** Linda E Bröker, Gerard JB Hurenkamp, Gerben ter Riet, François G Schellevis, Hans G Grundmeijer, Henk C van Weert

**Affiliations:** 1 Department of General Practice, Academic Medical Center, University of Amsterdam, PO Box 22700, 1100 DE Amsterdam, The Netherlands; 2 Netherlands Institute for Health Services Research (NIVEL), PO Box 1568, 3500 BN Utrecht, The Netherlands; 3 Department of General Practice/EMGO institute, VU University Medical Centre, Postbus 7057, 1007 MB Amsterdam, The Netherlands

## Abstract

**Background:**

The pathophysiology of upper gastrointestinal (GI) symptoms is still poorly understood. Psychological symptoms were found to be more common in patients with functional gastrointestinal complaints, but it is debated whether they are primarily linked to GI symptoms or rather represent motivations for health-care seeking. Purpose of our study was to compare co-morbidity, in particular psychological and social problems, between patients with and without upper GI symptoms. In addition, we investigated whether the prevalence of psychological and social problems is part of a broader pattern of illness related health care use.

**Methods:**

Population based case control study based on the second Dutch National Survey of general practice (conducted in 2001). Cases (adults visiting their primary care physician (PCP) with upper GI symptoms) and controls (individuals not having any of these complaints), matched for gender, age, PCP-practice and ethnicity were compared. Main outcome measures were contact frequency, prevalence of somatic as well as psychosocial diagnoses, prescription rate of (psycho)pharmacological agents, and referral rates. Data were analyzed using odds ratios, the Chi square test as well as multivariable logistic regression analysis.

**Results:**

Data from 13,389 patients with upper GI symptoms and 13,389 control patients were analyzed. Patients with upper GI symptoms visited their PCP twice as frequently as controls (8.6 vs 4.4 times/year). Patients with upper GI symptoms presented not only more psychological and social problems, but also more other health problems to their PCP (odds ratios (ORs) ranging from 1.37 to 3.45). Patients with upper GI symptoms more frequently used drugs of any ATC-class (ORs ranging from 1.39 to 2.90), including psychotropic agents. The observed differences were less pronounced when we adjusted for non-attending control patients. In multivariate regression analysis, contact frequency and not psychological or social co-morbidity was strongest associated with patients suffering from upper GI symptoms.

**Conclusion:**

Patients with upper GI symptoms visit their PCP more frequently for problems of any organ system, including psychosocial problems. The relationship between upper GI symptoms and psychological problems is equivocal and may reflect increased health care demands in general.

## Background

Upper gastrointestinal (GI) symptoms are common complaints affecting 25-40% of the general population during their lifetime [1,2]. Organic disorders such as peptic ulcers and gastro-esophageal reflux disease only account for a minority of cases, and in most patients no cause is found. These functional upper gastrointestinal symptoms, comprising dyspepsia, heartburn, epigastric discomfort and other abdominal complaints, are classified by ROME III criteria . They are never life threatening, but represent major burdens on health care services [2,3] and quality of life [4,5]. Despite their important medical and economic implications, the pathophysiologic mechanisms involved in functional gastrointestinal symptoms are still poorly understood. Traditionally, psychological factors were held responsible for upper GI symptoms [6]. With the identification of *Helicobacter Pylori *the etiological paradigm changed dramatically, but eradication therapy proved to be of only limited value in functional dyspepsia [7]. Several mechanisms such as visceral hypersensitivity and altered brain-gut interactions have been postulated to play an etiological role in dyspepsia (reviewed in [8,9]). In recent years, there has been a renewed interest in psychological factors in the pathophysiology. Interestingly, symptoms of neurosis, anxiety, hypochondria and depression were found to be more common in patients with unexplained gastrointestinal complaints when compared to controls. It remains, however, matter of debate whether psychological factors are causal to functional dyspepsia or whether they are linked to increased health care demands in general (reviewed in [10]).

In 2001, a National Survey of General Practice was conducted among over 100 practices in the Netherlands investigating health problems and contacts with the primary care physician (PCP) of 400,000 inhabitants during a period of one year. This survey offered us the unique opportunity to conduct a population-based case control study on psychological and social co-morbidity and health care demands in patients with upper GI symptoms. Aim of our study was to investigate whether psychological and social problems are more frequent in patients with upper GI symptoms and whether their prevalence is part of a broader pattern of illness related health care use.

## Methods

### Second Dutch National Survey of General Practice

The study took place in the framework of the second Dutch National Survey of General Practice conducted by NIVEL (Netherlands Institute for Health Services Research) in 2001. In this survey, 195 PCPs in 104 practices participated. Because of insufficient quality of data registration, eight of the participating practices were excluded from analysis. In the Netherlands, virtually all inhabitants are registered with a PCP, who acts as a gatekeeper to secondary care. The listed mid-time population size of this national survey was 375,899, comprising a 2.4% sample of the Dutch population. The patient population was representative for the Dutch population with respect to age, gender, social class, degree of urbanization, ethnic minority groups and type of health insurance [11]. The participating PCPs were representative for Dutch PCPs with respect to age, gender and location in deprived areas. However, single-handed practices were relatively underrepresented (32% instead of 44% nationwide [11]).

Study design and methods of the survey have been published in more detail elsewhere [11]. In short, for a period of one year data about all patient contacts, including diagnoses, referrals and prescriptions, were coded by all participating PCPs. All diagnoses were coded according to the International Classification of Primary Care version 1 [12] and prescriptions were coded according to the Anatomical Therapeutic Chemical (ATC) Classification system. Different health problems within one consultation were recorded separately.

### Selection of patients and controls

In order to provide optimal generalization to general practice, we did not only include dyspepsia, but used a broad definition of upper GI symptoms including epigastric pain, heartburn, esophageal disease, stomach function disorders and peptic ulcers. Patients with upper GI symptoms were selected from the overall study-population under the condition that they were registered by their PCP with at least one of the following diagnoses during the registration period: epigastric abdominal pain (D02), heartburn (D03), esophageal disease (D84; including esophageal diverticulum, esophagitis and reflux esophagitis), duodenal ulcer (D85), other peptic ulcer (D86; including gastric ulcer and other peptic ulcers), stomach function disorder (D87; including gastritis/duodenitis, dyspepsia and other stomach function disorders). In the second Dutch National Survey, outcome of endoscopic investigations or laboratory results were not separately registered. Control patients were randomly selected if they did not have any of the above diagnoses in the period of registration, and were individually matched (1:1) on practice, gender and age (± 2 years, if not possible ± 5 years). As dyspeptic complaints are known to be influenced by ethnic origin [2], control patients were also matched on ethnicity. Ethnic groups were defined according to Statistics Netherlands as native Dutch, Western immigrant (Europe (excluding Turkey), North-America, Oceania, Israel, Japan or Indonesia) and non-Western immigrant (Turkey, Asia (excluding Japan and Indonesia), Central- and South America, Africa). We selected adult patients (≥ 18 years) for all analyses.

### Outcome measures

Primary outcome measure was the prevalence of diagnoses in the various ICPC-chapters. Psychosocial co-morbidity was defined as the prevalence of one or more diagnoses in the 'psychological' and/or 'social' ICPC-chapter. Secondary outcome measures were prescription rates at ATC main class-level, prescription rates of psychopharmacological agents (defined as anxiolytic agents (N05B), sedatives and hypnotic agents (N05C) and antidepressants (N06A)), contact frequency and referral rates to secondary care. The latter two secondary outcome measures defined 'health care seeking'.

### Statistical Analysis

Missing values were only present for 'level of education' and 'ethnicity', and were multiply imputed using iterative chained equations [13,14]. We used descriptive statistics for baseline characteristics and odds ratios and the Chi square test to compare proportions between groups. We used the Student's *t*-test to test for differences in means. We used two-sided tests, and considered results significant when P < 0.01. We performed multivariable random effects logistic regression analysis to describe independent associations between patient characteristics (including matching variables) and dyspepsia thus accounting for any intercluster (that is: PCP practice) correlation. The continuous variable 'contact frequency' was categorized. We used SPSS for Windows (version 14.0.2) and STATA (version 10.1) for statistical analyses.

### Ethics approval

The study was carried out according to Dutch legislation on privacy. Dutch Data Protection Authority approved the privacy regulation of the study. Dutch legislation does not require informed consent for observational studies.

## Results

### Patient characteristics

A total of 13,389 adult persons were identified with at least one upper GI symptom during the period of registration. For 13,288 patients a control counterpart meeting all matching criteria could be selected, but 101 patients could not be matched on ethnicity. Table 1 summarizes the patient characteristics of the upper GI symptoms and control patients. In general, the control group was higher educated. In the group of patients with upper GI symptoms, 4,329 patients (32.4%) had epigastric abdominal pain, 2,583 patients (19.4%) had heartburn, 3,102 patients (23.2%) had esophageal disease, 437 (3.3%) had duodenal ulcer, 255 patients (1.9%) had an other peptic ulcer and 4300 patients (32.2%) had a stomach function disorder (data not shown). In total, 88.8% of patients had one upper GI symptom, 10.1% had two and 1.1% of patients had three or more upper GI symptoms (range 1-5; median 1). By definition, none of the control patients had any of these diagnoses.

**Table 1:** Patient characteristics

	**Upper GI symptoms**	**Control patients**
**Number of patients**	13,389	13,389

**Sex **(n (%))		

Male	5,965 (44.6)	5,965 (44.6)

Female	7,424 (55.4)	7,424 (55.4)

**Age **(years)		

Mean	53.3	53.4

Range	18 - 100	18 - 98

**Ethnicity **(n (%))		

native Dutch	9,214 (68.8)	9,280 (69.3)

Western immigrant	721 (5.4)	692 (5.2)

non-Western immigrant	850 (6.4)	778 (5.8)

unknown	2,604 (19.4)	2,639 (19.7)

**Level of Education **(n (%))		

low^†^	3,218 (24.0)	2,688 (20.1)

intermediate^†^	6,041 (45.1)	6,097 (45.5)

high^†^	1,350 (10.1)	1,754 (13.1)

unknown	2,780 (20.8)	2,850 (21.3)

^†^low = primary school or no education; intermediate = high school; high = at least college

### Co-morbidity

As presented in Table 2, patients with upper GI symptoms more frequently had diagnoses of any organ system, including psychological and social problems, than control patients. In the control group, 2,306 patients (17%) did not contact their PCP during the period of registration, whereas in the upper GI symptoms group all patients had, by definition, at least one PCP-contact. When we adjusted for non-attending patients, the differences between the control group and the patients with upper GI symptoms became smaller, but remained statistically significant. Odds ratios then ranged from 1.12 (95% CI 1.02 to 1.24) for the ICPC-chapter 'pregnancy and family planning' to 1.80 for psychological diagnoses (95% CI 1.66 to1.91) and 2.77 (95% CI 2.60 to 2.95; data not shown) diagnoses in the digestive system, excluding diagnoses defining the upper GI symptoms group. The most frequent psychological and social diagnoses were similar for patients with and without upper GI symptoms and the most frequently presented health problems were overlapping for both patient groups when upper GI symptoms were not taken into account (data not shown).

**Table 2:** Proportion of patients with a diagnosis in one of the ICPC chapters

	**Upper GI symptoms** **(n = 13,389)**	**Control patients** **(n = 13,389)**	**Odds ratio** **(95% CI)**
**General and unspecified**	29.9	17.1	2.07 (1.95-2.19)

**Blood**	5.1	2.9	1.81 (1.60-2.06)

**Digestive**	100	13.0	-^‡^

Excluding D02-03, 84-87^†^	34.0	13.0	3.45 (3.25-3.67)

**Eye**	13.0	8.5	1.61 (1.49-1.74)

**Ear**	14.9	10.7	1.45 (1.35-1.56)

**Cardiovascular**	34.6	24.8	1.61 (1.53-1.70)

**Musculoskeletal**	54.4	35.6	2.16 (2.06-2.27)

**Neurological**	16.3	9.2	1.92 (1.78-2.07)

**Psychological**	27.5	14.4	2.25 (2.11-2.39)

**Respiratory**	45.1	30.5	1.86 (1.78-1.97)

**Skin**	38.7	27.1	1.70 (1.61-1.79)

**Endocrine/metabolic and nutritional**	17.0	11.8	1.53 (1.43-1.64)

**Urological**	14.6	8.4	1.86 (1.72-2.02)

**Pregnancy, family planning**	7.9	5.9	1.37 (1.25-1.51)

**Female genital**	16.2	11.5	1.49 (1.39-1.60)

**Male genital**	4.3	2.8	1.59 (1.39-1.82)

**Social problems**	6.4	3.5	1.88 (1.68-2.11)

**ICPC code unknown**	59	44.4	1.80 (1.72-1.89)

^†^diagnoses defining dyspepsia-patient group; ^‡^no odds ratios (OR) could be calculated because a diagnosis in the digestive system was the selection criterion

### Prescription of (psycho) pharmacological agents

As presented in Table 3, PCPs prescribed more psychotropic drugs to patients with upper GI symptoms than to control patients. These higher prescription rates were observed for anxiolytic, sedative as well as for antidepressant agents. However, the percentage of patients with a prescription during the period of registration was higher for all ATC-main classes in the group of patients with upper GI symptoms compared to the controls (Table 4). This was reflected in the general prescription rate. Patients with upper GI symptoms had a mean number of prescriptions of 16.4 (95% CI 16.1 to16.8), versus 8.4 in the control group (95% CI 8.2 to 8.7). When drugs for acid related disorders were excluded, this difference was slightly less pronounced (mean number of prescriptions in the group with upper GI symptoms 13.7, 95% CI 13.4 to 14.0).

**Table 3:** Proportion of patients with a prescription for psychopharmacological agents.

	**Upper GI symptoms** **(n = 13,389)**	**Control patients** **(n = 13,389)**	**Odds ratio** **(95% CI)**
**Psychotropic drugs**^†^	31.5	17.2	2.21 (2.09-2.34)

**Anxiolytic agents **(N05B)	17.5	8.7	2.22 (2.07-2.40)

**Sedatives and hypnotic agents **(N05C)	12.9	7.3	1.89 (1.74-2.05)

**Antidepressants **(N06A)	11.9	5.9	2.15 (1.96-2.35)

^†^including anxiolytic agents, sedative/hypnotic agents and antidepressant drugs

**Table 4:** Proportion of patients with a prescription in one of the ATC main classes

	**Upper GI symptoms** **(n = 13,389)**	**Control patients** **(n = 13,389)**	**Odds ratio** **(95% CI)**
**Alimentary tract and metabolism**	92.5	17.3	59.1 (54.7-63.9)

Excluding drugs for acid related disorders (A02^†^)	32.3	14.1	2.90 (2.73-3.08)

**Blood**	18.5	13.2	1.50 (1.40-1.60)

**Cardiovascular system**	34.2	25.6	1.51 (1.44-1.59)

**Dermatologicals**	32.8	21.4	1.79 (1.70-1.89)

**Genito-urinary system and sex hormones**	20.4	15.6	1.39 (1.31-1.48)

**Systemic hormonal preparations excluding insulin**	10.0	6.6	1.59 (1.45-1.73)

**Anti-infectives**^‡^	42.1	30.5	1.66 (1.58-1.74)

**Antineoplastic and immunomodulating agents**	1.1	0.8	1.44 (1.12-1.85)

**Musculoskeletal system**	33.9	21.6	1.86 (1.76-1.96)

NSAIDs* (M01A^†^)	31.5	20.0	1.83 (1.73-1.94)

**Nervous system**	45.1	26.0	2.34 (2.22-2.46)

**Antiparasitic products, insecticides, repellents**	1.9	1.0	1.97 (1.59-2.43)

**Respiratory system**	32.1	20.4	1.84 (1.74-1.95)

**Sensory organs**	14.8	10.1	1.55 (1.44-1.67)

**Various**	0.2	0.1	1.93 (1.01-3.68)

^†^ATC-code; ^‡^including influenza vaccines; *non-steroid anti-inflammatory drugs

### Psychosocial co-morbidity and health care seeking

The mean number of PCP-contacts of patients with upper GI symptoms was with 8.6 (95% CI 8.5 to 8.8) almost twice as high as compared to controls (mean number of contacts 4.4; 95% CI 4.3 to 4.5). This difference was slightly less pronounced when we corrected for non-attending control patients (mean number of contacts 5.3; 95% CI 5.2 to 5.4). In addition, more patients with upper GI symptoms were referred to a specialist compared to control patients: 42% of the patients had a referral to secondary care, versus 26% of the controls (OR 2.01, 95% CI 1.99 to 2.21). Table 5 presents the results of a multivariable logistic regression analysis. A diagnosis of upper GI symptoms is the dependent variable. Contact frequency was strongly associated with this group of patients, whereas psychological diagnoses were associated only weakly, and social problems not at all. Remarkably, endocrine diagnoses had an inverse relationship with upper GI complaints. Use of NSAIDs was not independently associated with the patient group.

**Table 5:** Patient characteristics and diagnoses independently associated with upper GI symptoms. The intercluster correlation coefficient (= PCP-practice) for this analysis is 0.00767 (95% CI 0.00382 - 0.0115).

**Variable**^†^	**Odds ratio (95% CI)**
**Contact frequency**	

4 -7/year (versus 0-3/year)	3.36 (3.11-3.63)

8-11/year (versus 0-3/year)	4.99 (4.50-5.54)

>12/year (versus 0-3/year)	6.29 (5.53-7.15)

**Digestive **(excluding D02-03, 84-87*)	2.03 (1.89-2.17)

**Musculoskeletal**^‡^	1.24 (1.17-1.31)

**Psychological**^‡^	1.22 (1.14-1.31)

**Neurological**^‡^	1.12 (1.03-1.22)

**General**^‡^	1.11 (1.03-1.19)

**Endocrine**^‡^	0.92 (0.85-0.99)

**Ethnicity**	

Native Dutch	Reference

Western immigrant	1.12 (1.00-1.25)

Non-Western immigrant	0.92 (0.82 - 1.04)

**Level of education**	

**Low**	1.20 (1.11-1.29)

**Intermediate**	Reference

**High**	0.87 (0.80-0.94)

**Sex**	

**Female**	Reference

**Male**	0.67 (0.63-0.71)

**Age **(per year)	0.983 (0.981 - 0.985)

^†^variables not reaching statistical significance: blood^‡^, eye^‡^, ear^‡^, respiratory^‡^, skin^‡^, urological^‡^, pregnancy/family planning^‡^, male genital^‡^, female genital^‡^, social problems^‡^, NSAID-use. ^‡^diagnosis in ICPC-subchapter during the period of registration. *diagnoses defining group of patients with upper GI symptoms

## Discussion

In this large population based case control study, we have observed that patients with upper GI symptoms visit their PCP more frequently with psychological and social problems than patients not having these complaints. In addition, PCPs prescribed more anxiolytic, sedative and antidepressant agents than to patients not having upper GI symptoms. However, the increased health care demands of patients with upper GI symptoms were not restricted to psychosocial problems, but comprised all organ systems. Patients with upper GI symptoms visited their PCP twice as often and received up to double the number of prescriptions in any ATC-class compared to control patients. We demonstrated that not psychological and social co-morbidity, but high contact frequency in general is most strongly associated with upper GI symptoms.

To the best of our knowledge, our study is the largest population-based survey reported to date addressing the issue of psychosocial co-morbidity, health care demands and upper GI symptoms. We used a broad definition including both organic and functional dyspepsia in order to reach an optimal generalization to the general practice population. Almost 100 PCPs participated in this second National Survey, which further increases the generalization of our findings. We did not use validated questionnaires to objectify PCP's diagnoses of psychological co-morbidity. Our study was based on cross sectional data and was not designed to demonstrate temporal or causal relationships.

Several case-control studies have been conducted investigating the relationship between functional dyspepsia and psychological distress. Anxiety, neuroticism, depression and life stress were found to correlate with functional dyspepsia [15-20]. Although the extrapolation of these studies is limited due to low patient numbers and/or selection of dyspepsia patients from secondary care settings, a large international population-based study confirmed that upper gastro-intestinal symptoms were associated with psychological stress [21]. Similar results were reported by Herschbach et al. who investigated a large sample (n = 2,201) of the German population. In the latter study, the psychopathology seen in people with functional gastro-intestinal disorders was reported to be of two types: one as a characteristic of the illness itself, whereas the other leads the individual to consult a physician [22]. This is in line with our findings that patients with upper GI symptoms more frequently have psychosocial morbidity, and present more health problems to their PCP.

Factors found to contribute to health care seeking in dyspeptic patients are frequent or severe symptoms, increasing age and lower socio-economic status [23-25]. Indeed, low level of education was independently associated with patients with upper GI symptoms in our study, but we observed an inverse association between age and upper GI symptoms. Other factors that may favor seeking medical attention are hypersensitivity to bodily conditions and an active coping style attempting to solve one's (medical) problems [26], psychological distress [27] and anxiety [28], but these findings could not be confirmed by others [24,25].

Although our study was not designed to demonstrate causal relationships, it is tempting to speculate on the relationship between upper GI symptoms, contact frequency and psychological co-morbidity. Patients who consult their PCP frequently because of their coping style and attentiveness to physical symptoms (or other, yet unidentified reasons) may just have a high chance to be diagnosed in any health domain, including the psychosocial. In other words, upper GI symptoms and psychosocial complaints may both be manifestations of increased health care demands and not etiologically related. Our finding that the most frequent diagnoses (overall as well as in the psychosocial subsections) were similar in both patient groups supports this hypothesis. In this regard it is interesting that endocrine diagnoses, which can be considered as objective diagnoses (assessed by laboratory results and therefore less dependant of interpretation by PCPs), not depending on psychosomatic factors, had an inverse association with the patient group in our study, also after adjustment for age. On the other hand, psychological distress and anxiety may provoke symptoms of many organ systems including the stomach that prompt patients to consult a physician. Although there is insufficient evidence for the efficacy of psychological interventions in functional dyspepsia [29], this theory implies that exploration of psychological problems may be beneficial for patients with upper GI symptoms.

The high prescription rates for patients with upper GI symptoms observed in our study involved not only acid suppressant drugs, but comprised all ATC classes. Although it is known that acid suppressant agents belong to the most frequently prescribed drugs , our study is the first to clearly demonstrate the polypharmacy of patients with upper GI symptoms. Our findings indicate that caution is needed when prescribing additional drugs to these patients, as the risk of adverse effects and unwanted drug-drug interactions increases with increased number of prescriptions [30].

## Conclusion

In conclusion, our study demonstrates that the increased health care demands of patients with upper GI symptoms go beyond dyspeptic complaints and psychological problems and involve almost all organ systems. Whether psychological distress is the common cause for the perceived ill health or whether psychological distress and upper GI symptoms are both signs of increased health care needs has to be determined in a well designed prospective study.

## List of abbreviations

ATC: Anatomical Therapeutic Chemical; CI: confidence interval; PCP: primary care physician; ICPC: International Classification of Primary Care; NSAID: non-steroid anti-inflammatory drug; OR: odds ratio.

## Competing interests

The authors declare that they have no competing interests.

## Authors' contributions

LB: analysis and interpretation of the data, drafting the manuscript. GH: conception and design of the case control study, interpretation of the data, critically revising the article. GtR: analysis and interpretation of the data, critically revising the article. FS: conception and design of the second dutch national survey, critically revising the article. HG: conception and design of the case control study, critically revising the article. HvW: analysis and interpretation of the data, critically revising the article. All contributors approved the final version of the manuscript.

## Pre-publication history

The pre-publication history for this paper can be accessed here:


